# A differential Hall effect measurement method with sub-nanometre resolution for active dopant concentration profiling in ultrathin doped Si_1−_*_x_*Ge*_x_* and Si layers

**DOI:** 10.3762/bjnano.9.184

**Published:** 2018-07-05

**Authors:** Richard Daubriac, Emmanuel Scheid, Hiba Rizk, Richard Monflier, Sylvain Joblot, Rémi Beneyton, Pablo Acosta Alba, Sébastien Kerdilès, Filadelfo Cristiano

**Affiliations:** 1 LAAS-CNRS and Univ. of Toulouse; 2 STMicroelectronics, 850 rue Jean Monnet, 38926 Crolles, France; 3 CEA-LETI and Univ. of Grenoble, 17 rue des Martyrs, 38054 Grenoble, France

**Keywords:** carrier mobility, contact resistance, differential Hall effect, dopant activation, fully depleted silicon on insulator (FDSOI), laser annealing, sub-nanometre resolution

## Abstract

In this paper, we present an enhanced differential Hall effect measurement method (DHE) for ultrathin Si and SiGe layers for the investigation of dopant activation in the surface region with sub-nanometre resolution. In the case of SiGe, which constitutes the most challenging process, we show the reliability of the SC1 chemical solution (NH_4_OH/H_2_O_2_/H_2_O) with its slow etch rate, stoichiometry conservation and low roughness generation. The reliability of a complete DHE procedure, with an etching step as small as 0.5 nm, is demonstrated on a dedicated 20 nm thick SiGe test structure fabricated by CVD and uniformly doped in situ during growth. The developed method is finally applied to the investigation of dopant activation achieved by advanced annealing methods (including millisecond and nanosecond laser annealing) in two material systems: 6 nm thick SiGeOI and 11 nm thick SOI. In both cases, DHE is shown to be a uniquely sensitive characterisation technique for a detailed investigation of dopant activation in ultrashallow layers, providing sub-nanometre resolution for both dopant concentration and carrier mobility depth profiles.

## Introduction

The research efforts made throughout the last decades have made it possible to keep the momentum for a continuous miniaturization of electronics devices. For instance, the “bulk” planar transistor limitations have been overcome thanks to the transition towards more complex device architectures. These include enhanced planar architectures such as fully depleted silicon on insulator (FDSOI) [1] or 3D architectures ranging from TriGate FinFETs [2] to gate-all-around NWFETs [3] and monolithic 3D CoolCube technology [4]. Despite their differences, some technological issues have emerged as a significant challenge for all of them, such as the need to reduce the contact resistance at the silicide/source–drain interface [5].

The increase of the active dopant concentration at the surface of the source/drain material (usually Si or SiGe) is a key factor for obtaining a resistance reduction [6], and several process solutions have been proposed to this purpose, involving advanced implanting or annealing techniques [7]. Within this context, the optimization of existing characterisation techniques for the measurement of dopant activation at the semiconductor surface (or the development of new ones) is therefore decisive for both the improvement of the fabrication processes and the calibration of the related technology CAD (TCAD) physical models.

For device architectures based on planar SOI substrates (such as FDSOI or 3D CoolCube), measurements of active dopant concentrations from “blanket wafer” experiments are still relevant for process and TCAD optimisation, which are in principle achievable thanks to several known 1D measurement techniques previously developed for dopant profiling. However, in the case of contact resistance optimisation, only the dopant concentration close to the surface is relevant, i.e., within the first few nanometres, while the SOI/SiGeOI substrates used in current technologies are extremely thin (top layer < 10 nm), making measurement techniques with sub-nanometre resolution necessary. 1D techniques based on small-angle bevel preparation (such as spreading resistance profiling (SRP) [8] or scanning capacitance microscopy (SCM) [9–10]) become extremely difficult to implement and control in view of such a small resolution. Thanks to the use of an AFM tip, 2D scanning spreading resistance microscopy (SSRM) has been shown to achieve sub-nanometre resolution [11–12]. However, in this technique, the carrier concentration is inferred from a resistivity profile under the assumption that carrier concentration varies ideally with mobility, which is not always the case, especially when a part of the dopant is not electrically active [13]. For this reason, reliable mobility and concentration profiling based on scanning probe techniques require a combination of resistivity measurements by SSRM with carrier concentration measurements by SCM [14]. Finally, capacitance-based techniques such as SCM or electrochemical capacitance voltage (ECV) [15], provide reliable values of carrier concentrations only in the absence of additional electrically active defects, which can affect the CV signal [16].

In contrast, differential Hall effect (DHE) profiling [17–18] can potentially meet all the requirements related to the precise measurement of dopant activation at the semiconductor surface. DHE relies on the iteration of etching process and conventional Hall effect measurements. The active carrier profile is therefore measured without any assumption about the magnitude of the carrier mobility. In addition, measurements are made by stripping the material in successive steps rather than bevelling the surface. The depth resolution of the final dopant concentration profile is therefore defined by the etch rate and indeed nanometric resolution has been successfully demonstrated for Si and Ge, applying oxidation processes such as anodisation [19] or oxidising chemistry [20–23]. Nevertheless, etching SiGe alloys with nanometric resolution is far more challenging considering that Si and Ge have different oxidation rates [24]. For this reason, reliable DHE measurements of doped SiGe layers have not been reported in literature. Finally, in all published DHE investigations, the removal rate is assumed to stay constant. However, even small variations in the removed thickness among nominally identical etch steps can strongly distort the final carrier concentration and mobility profiles.

In this paper, we present an enhanced differential Hall effect measurement method that allows to precisely determine the level of dopant activation close to the semiconductor surface for Si and SiGe. First, we detail the etching processes that we have developed for each semiconductor, with particular focus to the SiGe case, which constitutes the most challenging process. For both materials, our method includes a direct measurement of the removed thickness after each removal step, so to avoid averaging the etch rate and improve the accuracy of final calculated values. Then, we demonstrate the reliability of a complete DHE procedure on a dedicated SiGe test structure fabricated by CVD and uniformly doped in situ during growth. Finally, we will apply our DHE method to the investigation of dopant activation achieved by advanced annealing methods in two material systems: 6 nm SiGeOI and 11 nm SOI.

## Development of Etching Processes for Si_1−_*_x_*Ge*_x_* and Si

### Etching process for Si_1−_*_x_*Ge*_x_*

Different methods have been proposed in literature for the controlled etch of SiGe layers [25–27]. We first analysed the main characteristics of each solution in terms of the specific requirements related to their application for DHE measurements. In particular, (i) the solution must etch Si and SiGe simultaneously so that the SiGe stoichiometry is not modified; (ii) the solution must be strongly selective with respect to Si so to preserve the surrounding Si areas in Van der Pauw test structures; (iii) the solution must be chemically active for a relatively long period (about 1 day) so to be used for several “etch and measurement” cycles; (iv) the etch rate must be slow (ca. 1 Å·min^−1^) to allow for sub-nanometre resolution. Taking into account the above mentioned criteria, we therefore selected the one-step chemistry based on SC1 (NH_4_OH/H_2_O_2_/H_2_O 1:1:5), which oxidizes and removes both materials at the same time.

**Figure 1:**
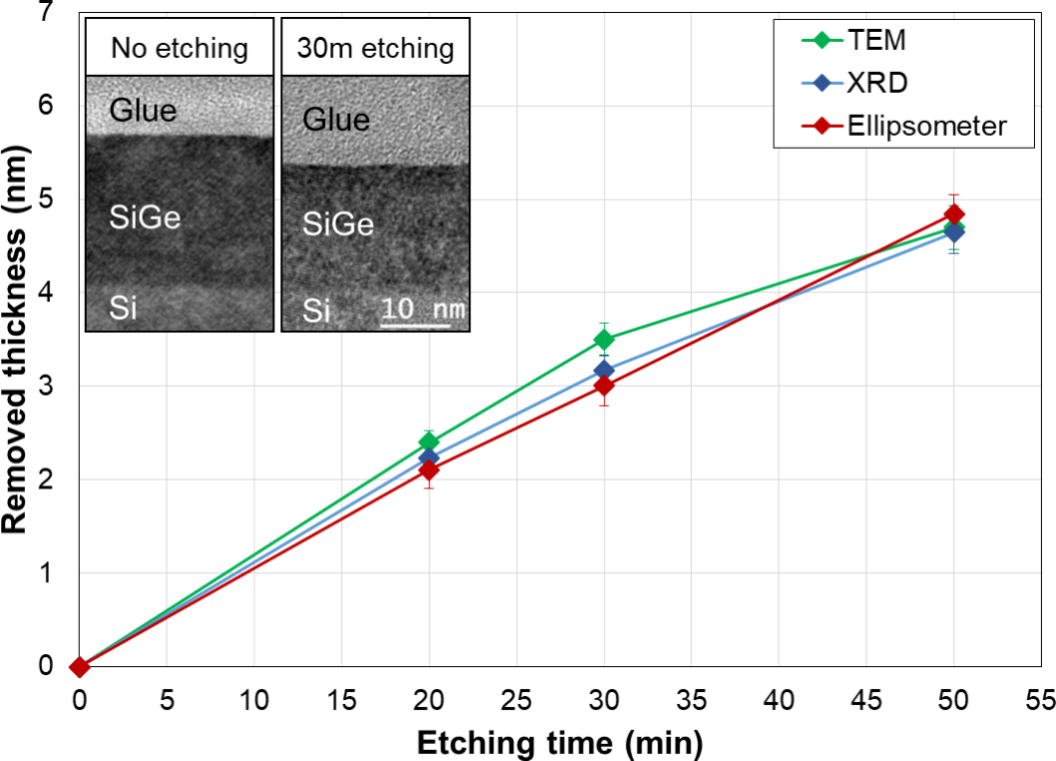
Removed SiGe thickness measured by different methods (TEM, XRD and ellipsometry) as a function of the etching time. Ge content: 27 atom %. Inset: TEM cross-section micrographs of reference and the sample etched for 30 min. This figure illustrates the agreement between the three chosen techniques.

We then investigated the efficiency of the SC1 solution by running several tests as a function of different experimental parameters including time, temperature and Ge content. For this, spectroscopic ellipsometry (with a HORIBA Jobin Yvon system) was used as a fast, reliable and non-destructive method for the measurement of the removed thickness. We developed an empirical model for the quantification of the SiGe thickness measurement (based on a SiGe/Si two layers stack and a point-by-point calculation procedure), which was calibrated using other techniques (such as TEM and XRD). As an example, Figure 1 summarizes the removed thickness measured by XRD (in (004) configuration), high-resolution TEM and ellipsometry as a function of the etching time of a 20 nm thick Si_0.73_Ge_0.27_ boron-doped layer (10^18^ cm^−3^) grown on top of a Si substrate.

TEM images show a clear decrease of the layer thickness, while all the techniques are in mutual agreement, therefore validating ellipsometry as a unique thickness characterization method for the remainder of this work. From this study we estimated a value of 0.95 Å·min^−1^ for the etch rate of the SC1 solution on Si_0.73_Ge_0.27,_ without any alteration of the initial layer stoichiometry, as confirmed by XRD analysis (Figure S1, Supporting Information File 1). Moreover, the found etch rate is in very good agreement with previous results obtained by our research group [26]. Concerning the surface roughness, tapping mode AFM analysis provided arithmetic averages *R*_a_ of about 1.2 Å (Figure S2, Supporting Information File 1).

**Figure 2:**
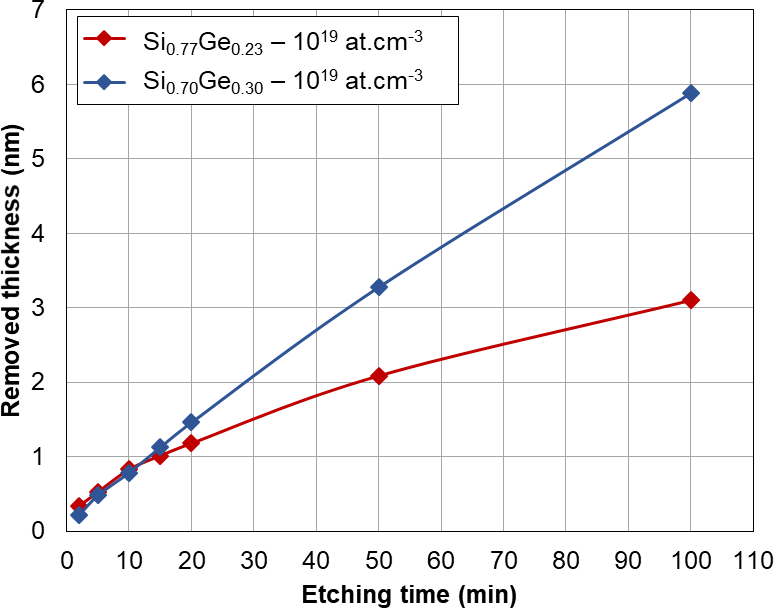
Removed SiGe thickness (measured by ellipsometry) as a function of etching time for two different Ge contents. After the first 15 min, the etch rate increases with Ge content.

However, in view of its application for DHE experiments, it is necessary to use an encapsulation cell to protect metallic contacts of the electric test structures during etch (Figure S3, Supporting Information File 1). Due to the funnel-shaped cell designed for this study, the reaction zone is confined, which results in a reduction of the etch rate. By optimising the experimental set up (use of a magnetic stirrer combined with an appropriate cell orientation in the solution bath), we managed to limit the etch rate reduction and similar values to experiments with “blanket” samples were found. Finally, we investigated the impact of the Ge content on the measured etch rate. The results are shown in Figure 2, where we compare the removed thickness as a function of the etching time for two 20 nm boron-doped (10^19^ cm^−3^) Si_1−_*_x_*Ge*_x_* samples with different germanium content : *x* = 0.22 and *x* = 0.30. For etching times less than 15 min, the etch rate is perfectly linear and independent of the Ge content, with a removed thickness of ca. 1 nm after 15 min. It is therefore possible to use this solution to achieve sub-nanometre resolution. In summary, all these investigations confirm the choice of SC1 as chemical solution for SiGe etching because of its slow etch rate, stoichiometry conservation and low roughness generation.

### Etching process for Si

The silicon etching process differs from the etching of SiGe insofar as it involves a two-step mechanism: first, oxidation and then oxide stripping. In this case, the etch rate is not defined as a function of the etching time, but is given by the removed thickness per step, i.e., the removed thickness between two stripping processes. A resolution of about 1 nm has been obtained in the study of Ling et al. [22] combining dilute HF, ultrapure water rinsing and re-oxidation in a clean-room environment. However, with the aim of minimising the surface roughness, we used ethanol instead of ultrapure water as rinsing solvent [28]. We performed multiple cycles of etching processes on in situ boron-doped Si layers (grown on top of Si substrates) with continuous monitoring of the removed thickness (by ellipsometry measurements) and the surface roughness (by AFM characterization). Our results show a cycle-by-cycle etch rate below 1 nm and a final roughness of 1 Å.

### DHE procedure validation on SiGe layers fabricated by CVD

In this section, we detail a complete DHE procedure using a 20 nm thick boron-doped (10^19^ cm^−3^) Si_0.77_Ge_0.23_ layer grown by CVD on top of a Si substrate. We first describe the Van der Pauw structure and the conventional Hall effect setup. Then we will present the differential Hall effect measurements and calculations and we will discuss the limitations of the technique.

### Van der Pauw structure and Hall effect measurements on ultrathin layers

The Hall effect measurement is a well-known technique that allows one to access three important physical parameters for material characterization: the sheet resistance *R*_s_, the active Hall dose *N*_H_ and the Hall mobility µ_H_. At first, a Van der Pauw technique is used to determine the sheet resistance, then a magnetic field is applied orthogonally to the sample surface to measure the sheet Hall coefficient *R*_SH_, which is finally used to deduce *N*_H_ and µ_H_.

Several classical Van der Pauw shapes were tested to perform electrical measurements (square, Greek cross and bridge “bar-shaped” structures). A test structure in the form of a Greek cross has been chosen as it has more advantages than other shapes (Figure S4, Supporting Information File 1). First, it provides an error of less than 1% on both sheet resistance and Hall coefficient measurements [29–32]. Moreover, it has a highly symmetrical shape with peripheral contacts separated from the centre region, in which the current lines converge allowing precise characterization. For this last reason, we were able to design an encapsulation cell (Figure S3, Supporting Information File 1) defining a reaction region in the centre part of the Greek-cross structure while protecting the metallic contacts with the lowest impact on structure symmetry and measurement reproducibility.

Electrical measurements were carried out with a HL5500PC Nanometrics Hall bench equipped with a 0.3 T magnet. For each investigated sample, the sheet resistance and the Hall coefficient were measured for several values of the injected current (from 1 μA to 1 mA), and the average values were determined within the current interval exhibiting the most stable measurements (Figure S5, Supporting Information File 1), so to keep the experimental errors close to 0.1%.

Scattering correction must be accounted for when extracting Hall effect parameters. The measured values of Hall carrier concentration and Hall mobility are therefore corrected by using the Hall scattering factor, *r*_H_, [33–35] which depends on the studied material, i.e., on Ge content, doping type and concentration. For this study, we used a set of dedicated test samples consisting of 20 nm thick epitaxially grown Si and SiGe layers, in situ doped with boron (from 1 × 10^18^ cm^−3^ to 1 × 10^20^ cm^−3^). By comparing experimental Hall values with average calculated values based on the dopant concentration profiles measured by SIMS, we determined a scattering factor of 0.75 for holes in Si and values ranging from 0.4 to 0.35 for holes in SiGe with a Ge content of 22 atom % and 30 atom %, respectively, in perfect agreement with literature (Figure S6, Supporting Information File 1) [33–35].

Some other possible limitations should be considered in view of the implementation of a DHE methodology on ultrashallow layers. One is quantum confinement, which has been shown to induce band modifications in ultrathin SOI layers with thicknesses close to ca. 3 nm [36]. However, the SOI and SiGeOI layers to be investigated in this work will have a minimum thickness of about 6 nm, so that the quantum-confinement effect can be neglected. An additional low-dimension effect is the dielectric confinement, which has been investigated in silicon nanowires surrounded by a dielectric material (such as its native oxide) [37–38]. For nanowire diameters of about 10 nm, a dopant deactivation is observed due to the dielectrical mismatch between the silicon and its surroundings. However, our previous investigations on 5 nm thick SiGeOI layers doped by ion implantation and activated by conventional rapid thermal annealing (RTA) [39–40] indicated a perfect correlation between measured activation and simulated activation, suggesting that dielectric confinement affects more significantly 3D than 2D structures at low dimensions.

Finally, when quantifying the active dopant and mobility depth profiles with DHE, the surface-depletion effect should be considered [41–42]. This results from carriers becoming trapped in surface states and can lead to a depletion of carriers below the surface. As a consequence, the DHE profile might require a correction (depth-scale translation) corresponding to the depletion width. And in the case of non-uniform doping profiles, the depletion width (and the related correction) will vary with depth. For example, in the particular case discussed in this section, the investigated 20 nm thick SiGe layer is uniformly doped at 10^19^ cm^−3^. For typical silicon-dioxide charge densities of 10^12^ cm^−2^·eV^−1^, simple calculations provide a depletion width of about 2 nm. Consequently, in this case, a depth-scale translation is necessary. However, for the higher carrier concentrations typically investigated in source/drain doping studies, such as those discussed in the next section (10^20^ cm^−3^ and above), and considering the same typical silicon-dioxide charge densities, the surface depletion is well below 1 nm (about 0.4 nm at 10^20^ cm^−3^ and less than 0.2 nm at 5 × 10^20^ cm^−3^; Figure S7, Supporting Information File 1) and its impact on the quantification of the DHE depth profiles can therefore be neglected.

### Differential Hall effect data measurements and limitations

**Figure 3:**
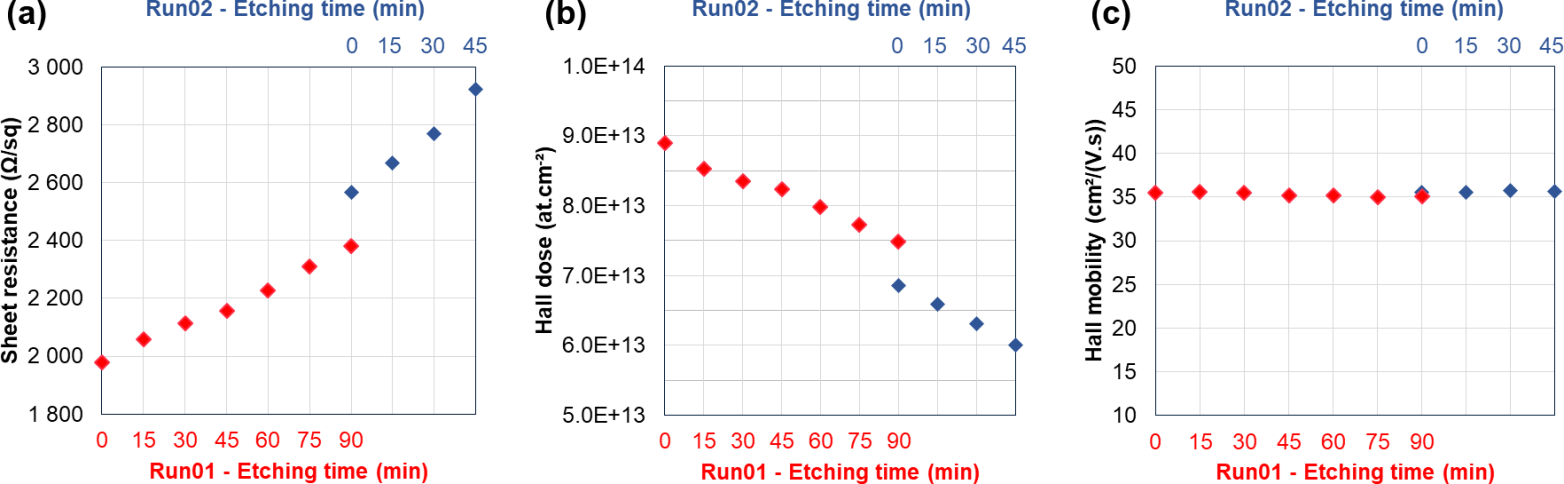
(a) Sheet resistance *R*_S_, (b) Hall dose *N*_H_, and (c) Hall mobility µ_H_ as functions of the etching time for a 20 nm thick SiGe layer (*x*_Ge_ = 0.23) grown by CVD and in situ doped with boron.

We performed a full set of DHE measurements on a 20 nm thick Si_0.77_Ge_0.23_ layer grown by CVD on top of Si a substrate and uniformly doped with boron at 10^19^ cm^−3^ (Figure S8, Supporting Information File 1). The layer was verified to be fully electrically active. A first run of six etch cycles (15 min each) was initially performed. The sample was then kept for three days in a clean room environment. Then, a second run of three etch cycles was carried out. Both runs were initiated without removing the initial native silicon dioxide. Electrical parameters *R*_S_, *N*_H_ and µ_H_ are reported in Figure 3 as a function of the etching time. Error bars are not reported as variations for each measured parameter are close to 0.1% (Figure S5, Supporting Information File 1).

Two different effects are observed. On one hand, the mobility stays constant with no discontinuity throughout the two measurement runs. On the other hand, the sheet resistance *R*_S_ constantly increases (while the Hall dose *N*_H_ decreases) and exhibits a discontinuity between the two runs. Indeed, as the doping concentration is uniform throughout the doped layer, the associated carrier mobility is expected to remain invariant in the entire layer. In contrast, as the layer becomes thinner and thinner, the active Hall dose decreases and, for a fixed carrier concentration (and hence mobility), the increase of the sheet resistance is predicted by Equation 1:

[1]
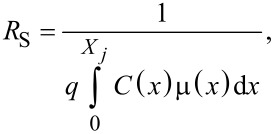


with *X_j_* being the layer thickness, *q* the electronic charge, *C*(*x*) the dopant concentration as a function of depth and µ(*x*) the corresponding mobility profile. The quasi-linear evolution of both *R*_S_ and *N*_H_ is therefore due to the combination of a uniform concentration profile and constant etching time intervals. Concerning the observed discontinuities, it must be considered that a regrowth of native oxide occurs between the last measurement of the first run and the first measurement of the second one. This regrowth reduces the SiGe thickness by about 1 nm (as measured by ellipsometry), which results in a sheet resistance increase and a decrease of the active Hall dose, without influencing the mobility.

Starting from these raw data, it was finally possible to calculate the differential values of the active concentration and mobility as a function of the depth. For the *i*-th etched layer, the calculated values are defined by the following equations [16]:

[2]
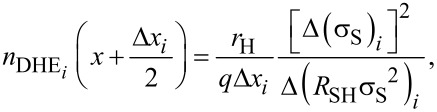


[3]
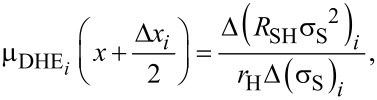


with


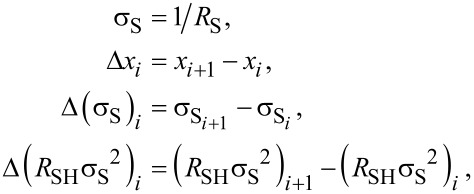


where the conductivity σ_S_ is given by inversing the measured values of the sheet resistance *R*_S_, and *R*_SH_ is the sheet Hall coefficient used to extract the Hall dose and carrier mobility for each measurement. The term Δ*x_i_* corresponds to the removed thickness after each etching process, which is determined by ellipsometry.

**Figure 4:**
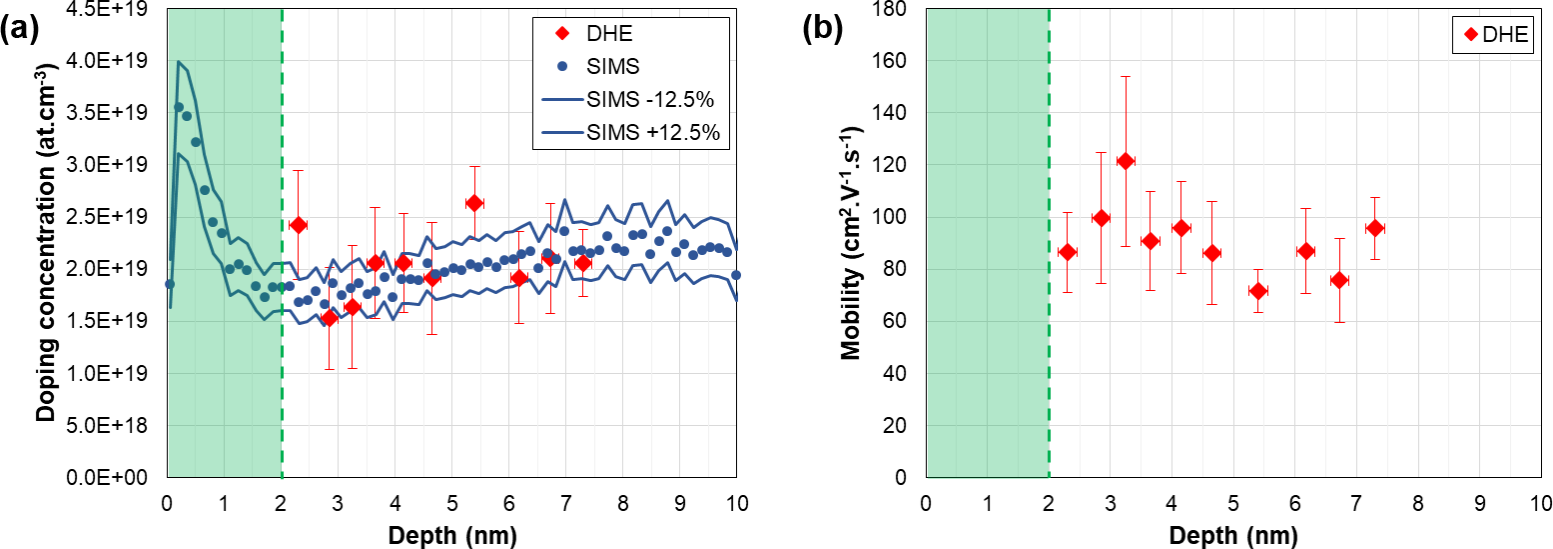
Depth profiles of (a) active dopant concentration and (b) carrier mobility extracted from the DHE measurements of Si_0.77_Ge_0.23_ uniformly boron-doped at 10^19^ cm^−3^. In panel (a), the active dopant concentration profile is compared to the chemical boron concentration profile measured with SIMS. Green areas are depletion regions.

From Equation 2 and Equation 3, DHE profiles of active dopant concentration and mobility are finally deduced and reported in Figure 4a and Figure 4b, respectively (red diamonds). The active dopant concentration profile is compared in Figure 4a with the chemical dopant profile measured by SIMS (blue dots). The comparison is made assuming a constant depletion width of 2 nm for each DHE measurement (in agreement with the uniform doping level of the investigated sample). Blue solid lines represent the possible error (±12.5%) of the SIMS concentration values quantified from standards. It has to be noted that the SIMS signal in the first nanometres below the surface is affected by measurement artefacts and cannot be considered as fully reliable. Also, at the beginning of each of the two measurement runs, the presence of a native oxide at the sample surface may result in a different electrostatic configuration of the surface compared to all other cases where the electrical measurements are performed just after the SC1 etching step. This is probably the reason for the upward shift of the calculated concentrations at the surface (first point in Figure 4a) and at a depth of 5.5 nm. Overall, Figure 4a shows a very good correspondence between the SIMS and the DHE profiles, in perfect agreement with the full electrical activation of the doped layer. More importantly, we show that the SC1 chemistry allowed us to achieve a depth resolution of ca. 0.5 nm.

The horizontal error bars of the DHE values are solely related to the uncertainty of the thickness measurements done by ellipsometry (with the surface-depletion effect having been accounted for by a rigid shift of the depth scale). Indeed, by performing ellipsometry measurements after each removal step, any possible source of errors related to etch rate variation during the experiment can be neglected. The vertical error bars uncertainties of DHE mobility and dopant concentration (*S*_µDHE_ and *S_n_*_DHE_, respectively) calculated assuming *R*_SH_, *σ*_S_ (and the product *R*_SH_·σ_S_^2^) to be independent variables [17]:

[4]
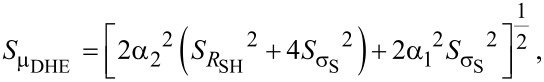


[5]
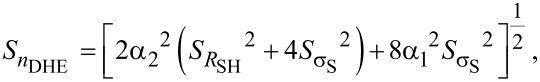


with

[6]
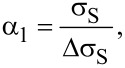


[7]
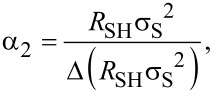


[8]
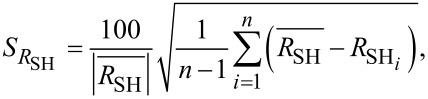


[9]
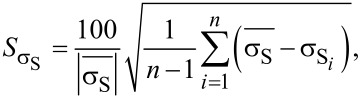


where *S*_RSH_ (Equation 8) and *S*_σS_ (Equation 9) represent the relative standard deviations of *R*_SH_ and σ_S_ calculated in the range of stability (Figure S5, Supporting Information File 1). It is interesting to note that for a chosen etching time interval, i.e., for a chosen depth resolution, α_1_ (Equation 6) and α_2_ (Equation 7) are constant. As a consequence, *S*_µDHE_ and *S_n_*_DHE_ can only be reduced by minimising *S*_RSH_ and *S*_σS_, in other words, by obtaining highly reproducible measurements of *R*_SH_ and σ_S_. One must therefore consider the importance of having reproducible measurements when performing DHE data reconstruction. Indeed, for a depth resolution of ca. 0.5 nm (as the one shown in Figure 4), targeted uncertainties of ca. 15% for µ_DHE_ and *n*_DHE_ requires that *R*_SH_ and σ_S_ must be measured with a relative standard deviation lower than 0.1%.

Within the experimental errors discussed above, the DHE mobility profile reported in Figure 4b gives a constant value of the mobility in the first 5 nm, in perfect agreement with the uniform nature of the concentration profile. The average value obtained through the calculated DHE points (with an etching step as small as 0.5 nm) is 91.02 ± 13.08 cm^2^·V^−1^·s^−1^, again in agreement with the more precise value of 88.60 ± 0.27 cm^2^·V^−1^·s^−1^ that can be extracted from the raw mobility data (cf. Figure 3c, *r*_H_ = 0.4) obtained from much thicker layers (between 15 and 20 nm thick). Also, these mobility values are perfectly compatible with those predicted by analytical models for a doping concentration varying between 1 × 10^19^ cm^−3^ (86 cm^2^·V^−1^·s^−1^) and 2 × 10^19^ cm^−3^ (74 cm^2^·V^−1^·s^−1^) with *x*_Ge_ = 0.23 at *T* = 300 K according to the following expression [43]:

[10]
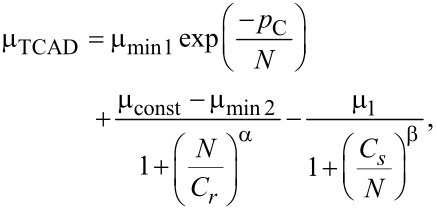


with


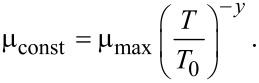


Considering the possible lack of precision in the Ge content of the layer as well as the sub-nanometric depth resolution achieved in these measurements, we can therefore conclude that the DHE method we have developed for the investigation of SiGe is consistent.

## Results and Discussion

### Study of a 6 nm boron-doped SiGeOI layer

Within the recent development of the 3D-sequential integration technology at CEA-LETI, laser annealing is being investigated as a low thermal budget solution for achieving dopant activation in the top transistor level without degrading the performance of the transistors located at the bottom [39]. The efficiency of this technique has already been proven for electrical activation of phosphorus in 22 nm thick SOI structures [44]. In this section, we extend the investigation to SiGeOI layers of 6 nm. Due to the extreme thin size of the layer and the buried oxide, classical 4PP characterization is not possible because of probe penetration down to the substrate. Thanks to Van der Pauw test structure, probe penetration has been circumvented, while conventional and differential Hall effect measurements described in the previous sections have been used to investigate dopant activation in laser-annealed ultrathin SiGeOI layers.

#### Experimental details

The starting SiGeOI wafer has a SiGe top layer of 6 nm and a 20 nm thick buried oxide (BOX). The first step is the deposition of a 3nm Si_3_N_4_ directly followed by Ge^+^ implantation to preamorphise a part of the SiGe crystal and B^+^ implantation for p-type doping. In the following step, a second layer of 3 nm Si_3_N_4_ is deposited prior to laser thermal annealing (LTA). LTA was performed by SCREEN-LASSE using a XeCl excimer laser (λ = 308 nm) with a pulse duration of approximately 160 ns. Finally, several 18 × 18 mm^2^ areas where irradiated with energy densities ranging from 0.65 to 0.79 J·cm^−2^ (Figure S9, Supporting Information File 1).

#### Structural and conventional Hall effect analysis

Prior to Hall effect analysis, we consider the structure of the layer before LTA. TEM cross-section observations (Figure S10, Supporting Information File 1) indicate that the top crystalline SiGe layer has a thickness between 5 and 6 nm, i.e., very close to the original thickness of 6 nm. Indeed, high-resolution images show that the layer thickness can rapidly vary by up to four lattice planes (i.e., ca. 1 nm) within a few nanometres. This suggests that the Ge preamorphisation implant in this wafer resulted in a damaged SiGe surface (locally amorphising it) but was not enough to produce a continuous amorphous layer.

Then, we compare electrical parameters measured by conventional Hall effect with the evolution of the crystal structure imaged by TEM as functions of the laser energy densities. The TEM analysis (Figure S11, Supporting Information File 1) shows that for energies of 0.74 and 0.76 J/cm^2^, the observed structure is identical to that found in the as-implanted sample with the SiGe layer being almost fully crystalline (and having a surface roughness of about 1 nm). This suggests that the laser energy density used in these cases is always lower than the threshold value necessary to melt the surface. In contrast, following a LTA at 0.79 J·cm^−2^ the SiGe top layer is completely amorphous, clearly indicating that in this case the whole SiGe layer was molten, leaving no seed for a perfect recrystallization. The threshold energy for surface melt is therefore located between 0.76 and 0.79 J·cm^−2^ and a rapid transition between a “no melt” and a “full melt” configuration occurs in this small energy interval.

**Figure 5:**
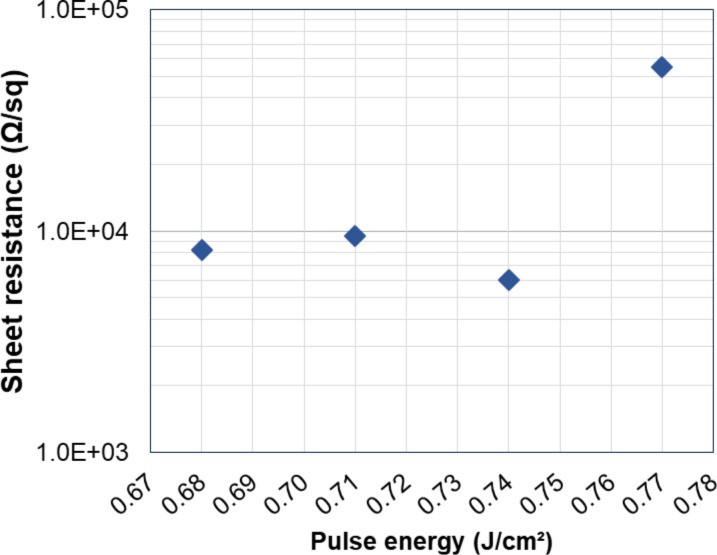
Sheet resistance as a function of the laser energy density for 6 nm SiGeOI (*x*_Ge_ = 0.25) layer implanted with boron.

Figure 5 reports the corresponding sheet-resistance measurements as a function of the energy densities, which illustrates two different behaviours. Below 0.74 J·cm^−2^, the sheet resistance remains below 10 kΩ·sq^−1^, with a slight improvement occurring when the energy density is increased (ca. 6000 Ω·sq^−1^ after LTA at 0.74 J·cm^−2^). This indicates that, although the laser annealing did not melt the sample surface, a non-negligible dopant activation occurs at these energies, as it will be discussed below. In contrast, a much higher sheet resistance value (ca. 55 kΩ·sq^−1^) is measured in the sample annealed at 0.77 J·cm^−2^. Considering that the transition between “no melt” and “full melt” of the 6 nm thick SiGe layer occurs between 0.76 and 0.79 J·cm^−2^, the high sheet resistance value measured at 0.77 J·cm^−2^ suggests that a “full melt” of the SiGe layer has already occurred at this energy and that most of the dopant activation is therefore lost. This behaviour is similar to that observed by Acosta Alba et al. [44] in 22 nm thick phosphorus-doped SOI, where the sudden increase in the sheet resistance values observed for high laser energies was due to the formation of a poly-Si layer as a consequence of the full melt of the entire top-Si layer during LTA.

For LTA energies below the melting threshold of 0.74 J·cm^−2^, some dopant activation occurs. However, the Hall effect measurements indicate that only a small fraction of the implanted boron dose is electrically active (between 6 and 12%). Two mechanisms contribute to this result: (i) the weak dopant penetration through the Si_3_N_4_ capping layer during the implantation, and (ii) the low activation rate due to the “non-melt” nature of the LTA in this energy range.

In order to investigate the first point, we calculated by using SRIM the depth distribution of the implanted boron ions according to the process conditions used in this experiment. The simulation results indicate that only about 45% of the implanted boron dose is available for electrical activation during LTA, the rest being lost in the Si_3_N_4_ capping layer or in the underlying BOX. Still, the boron dose contained in the SiGe layer after the implantation (ca. 1.8 × 10^14^ cm^−2^) is much higher than the electrically active dose actually measured by Hall effect (2.3 × 10^13^ cm^−2^ after LTA at 0.68 or 0.71 J·cm^−2^). In addition to this “dose loss” mechanism during implantation, low dopant activation must also occur during LTA.

Indeed, previous investigations [16,45] of dopant activation indicated that under similar conditions, i.e., non-amorphising implants and low thermal budget annealing (either conventional RTA or non-melt LTA), the total active dose (measured from SRP profiles) is much lower than the total implanted dose (as measured by SIMS profiles). However, the few electrically active dopant atoms present after annealing were not found to be uniformly distributed in depth but rather mostly located close to the surface, where the damage recovery, i.e., interstitial recombination is favoured (Figure S12, Supporting Information File 1). Moreover, even for the smallest thermal budgets (short RTA time or minimum number of laser shots), dopant activation at the surface was maximum or close to the solubility limit at the annealing temperature. Finally, it was found that dopant activation increases with annealing time although no dopant diffusion is detected by SIMS.

It is therefore important to verify if this behaviour also occurs in the case of ultrathin laser-annealed SiGeOI samples. Indeed, within the application of laser annealing in strategies to reduce contact resistance, such a result may constitute a big step forward. One of the LTA samples investigated in this work has therefore been analysed by the differential Hall effect technique, and results are presented in the next section.

#### Differential Hall effect analysis

The SiGeOI sample implanted with boron and annealed with an energy density of 0.68 J·cm^−2^ was used for these investigations. Four successive SC1 etching processes have been performed for a total etching time of 30, 50, 70 and 90 min, reducing, respectively, the total thickness by 0.1, 0.3, 0.8 and 1.3 nm (confirmed by ellipsometry measurements and TEM images). Concerning surface roughness, TEM images dos not show significant surface roughness, indicating that the surface quality is not degraded by the etch process. This was confirmed by AFM analysis on 500 **×** 500 nm^2^ areas taken from the Van der Pauw sample used for the Hall effect measurements after the longest etch process (90 min; Figure S13, Supporting Information File 1). Compared to the non-etched region (average roughness of 0.18 nm), the surface roughness is slightly higher in the etched regions (between 0.26 and 0.34 nm) but always much smaller than the total etched thickness (1.3 nm in this sample). Considering that these measurements were performed after the longest etch process and that the surface roughness increases with etching time, we can conclude that the surface roughness induced by the etch process is always negligible and is not expected to have any impact on the reliability of the Hall effect measurements.

**Figure 6:**
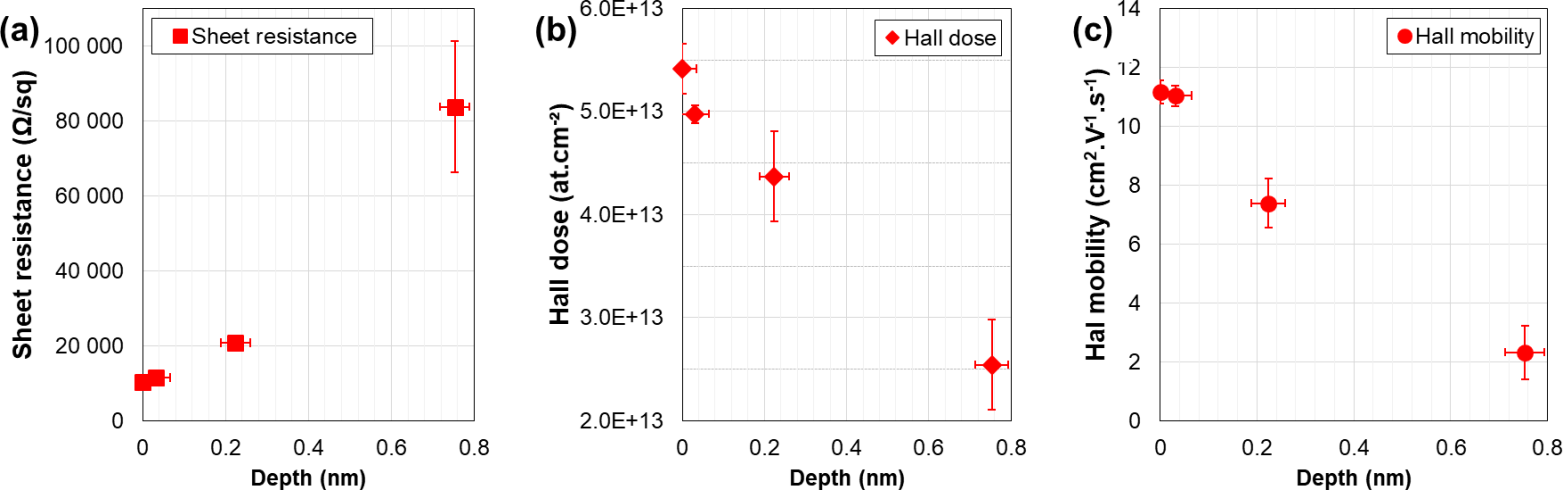
Hall effect measurements (raw data: (a) *R*_S_, (b) *N*_H_ and (c) µ_H_) of the SiGeOI sample (*x*_Ge_ = 0.25) implanted with boron and annealed at an energy of 0.68 J·cm^−2^ as a function of the etched thickness (as measured by ellipsometry).

The results of the Hall effect measurements (raw data *R*_S_, *N*_H_ and µ_H_) performed before etch and after each removal step are reported in Figure 6 as functions of the removed thickness. It appears that the sheet resistance *R*_S_ increases very rapidly after each step, with the *R*_S_ values increasing by a factor of four between the second and the third etch step. In fact, only the points corresponding to the three first etch steps are reported in the figure. Following the fourth and longest etch process (1.3 nm removed thickness) the sample was so resistive that quantitative values could not be measured. Correspondingly, the Hall dose *N*_H_ is found to rapidly decrease as the etching progresses, qualitatively indicating that most of the active dose is located close to the surface.

**Figure 7:**
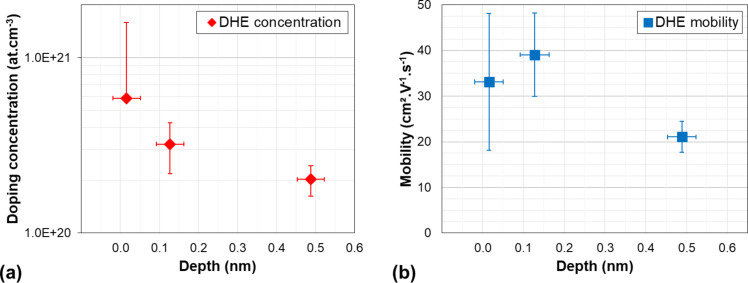
Depth profiles of (a) active dopant concentration and (b) carrier mobility extracted from DHE measurements of a SiGeOI sample (*x*_Ge_ = 0.25) implanted with boron and annealed at an energy of 0.68 J·cm^−2^.

Following the differential Hall data treatment method discussed in the previous sections, the depth distributions of the active dopant concentration and of the carrier mobility have finally been extracted and are reported in Figure 7. The data quantitatively confirm the results suggested by the Hall effect raw data: The active dopant concentration is highest at the surface with a value as high as ca. 6 × 10^20^ cm^−3^ and it rapidly decreases within the first nanometres below the surface (2 × 10^20^ cm^−3^ at 0.8 nm). Corrections of the depth scale related due to the surface-depletion effect have been neglected due to high doping level measured in this sample (cf. previous section) unless we give a numerical value.

This result is in agreement with the scenario discussed in the previous section. Indeed, due to the “non-melt” nature of the annealing, and considering that no amorphisation of the surface was achieved during the implantation, the extremely low thermal budget provided by the LTA process is not efficient in removing the implant damage in the material, except in the surface region where interstitial recombination (and hence damage recovery) occurs. As a consequence, below the surface, not only the active dopant concentration is much lower than at the surface, but also the residual damage is extremely high, which is expected to have an impact on the carrier mobility. This is clearly confirmed by the Hall mobility (Figure 7b) the value of which at a depth of 0.8 nm below the surface (ca. 20 cm^2^·V^−1^·s^−1^) is much lower than the carrier mobility at the surface (ca. 35 cm^2^·V^−1^·s^−1^), in spite of a much lower carrier concentration. Alternative mechanisms as the reasons for this mobility reduction below the surface can be excluded, including surface roughness (Figure S13, Supporting Information File 1), and surface depletion due to interface states (cf. previous sections).

In any case, although the investigated doping process is at a preliminary stage, the detailed investigation carried out in this work allows us to conclude that a doping process based on nanosecond-laser annealing can be successfully applied to ultrathin SiGeOI layers of about 6 nm thickness, while obtaining active dopant concentrations at the surface well above 1 × 10^20^ cm^−3^. This is a promising result in view of improving contact resistivity in source/drain regions of advanced devices.

### Study of 11 nm arsenic-doped SOI layer

In the perspective of improving the contact resistance within FDSOI technology [6], different annealing methods are investigated for the increase of dopant activation close to the surface. In this section we will focus on the comparison between conventional spike-RTA and millisecond-laser dynamic surface annealing (DSA), both applied to 11 nm thick n-type doped SOI layers. In addition to SIMS, TEM and conventional Hall effect measurements, differential Hall profiling will be shown to allow a reliable estimation of the dopant activation level within the first nanometres below the silicon surface.

#### Experimental details

Two 11 nm thick SOI wafers were used for this experiment (BOX thickness: 25 nm, as confirmed by ellipsometry). The wafers were implanted with 3 keV As^+^ ions to a dose of 1 × 10^14^ cm^−2^. The implantations were performed through a thin thermal oxide layer (ca. 1 nm thick) grown on the as-received wafers. Following the implantations, each wafer underwent a different annealing process: 1050 °C spike-RTA in O_2_/N_2_ atmosphere in one case, 0.3 ms laser-DSA in N_2_ atmosphere in the other case.

#### Chemical profiles and conventional Hall effect measurements

**Figure 8:**
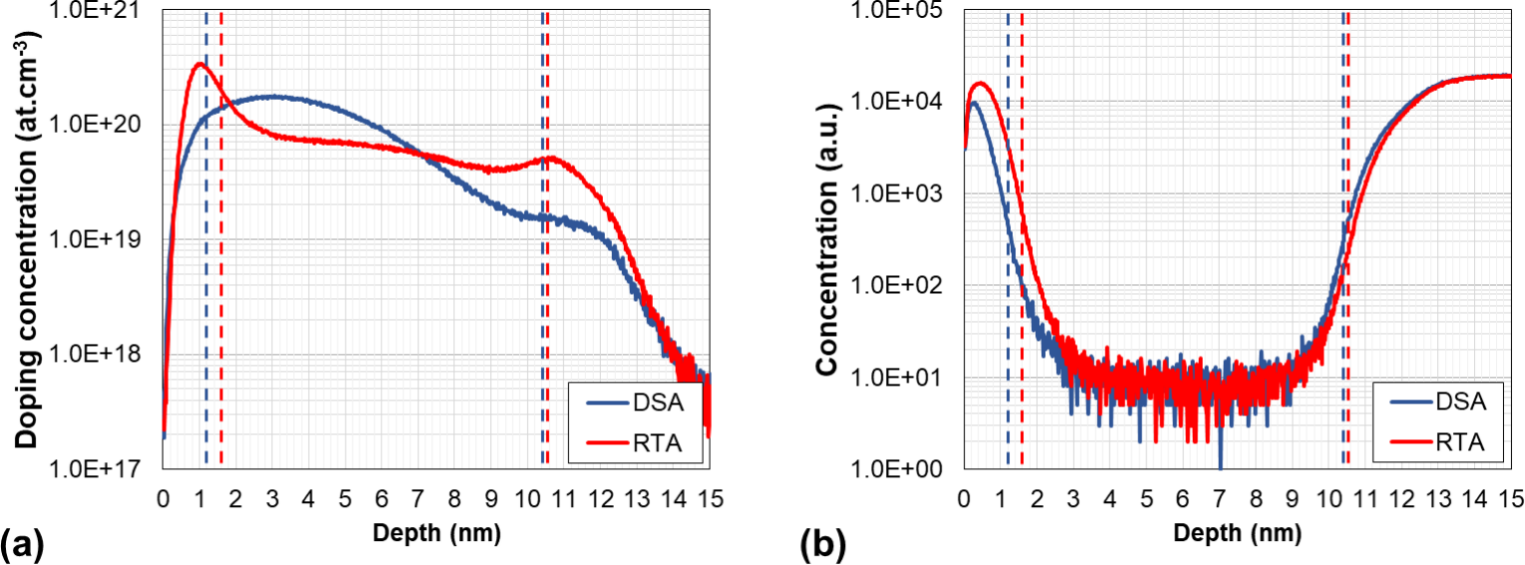
Concentration depth profiles of (a) arsenic and (b) oxygen measured by SIMS in 11 nm thick SOI wafers implanted with arsenic and annealed by RTA (red curves) or DSA (blue curves).

SIMS characterisations were carried out after annealing in both samples without stripping the thermal oxide. The results are shown in Figure 8 for both arsenic (Figure 8a) and oxygen (Figure 8b). The As concentration profile of the RTA-annealed wafer exhibits a peak value of ca. 3 × 10^20^ cm^−3^ just below the surface, followed by a quasi-plateau (6–7 × 10^19^ cm^−3^) in the rest of the Si top layer. In contrast, the As profile of the DSA wafer is closer to a Gaussian shape, with a peak concentration of about 2 × 10^20^ cm^−3^ at a depth of ca. 3 nm. In both cases, the sharp decrease of the As concentration below 11 nm corresponds to the transition from the Si top layer to the buried oxide. Similarly, the As signal in the first nanometres below the surface originates from the dopant atoms contained in the thermal oxide formed prior to the implantation. It is therefore critically important to localise the actual position of the oxide/top Si interface in view of the reliable interpretation of the Hall effect data in terms of dopant activation efficiency, i.e., estimation of the active dopant fraction. To this purpose, we used the oxygen SIMS concentration profiles (Figure 8b) to estimate the position of the oxide/top Si and of the top Si/BOX interfaces in correspondence of the maximum slope of the oxygen signal. The position of the SiO_2_/Si interfaces determined in this way (1.2 nm and 1.6 nm below the surface for the DSA- and RTA-annealed wafers, respectively) are in perfect agreement with those found by STEM-EDX measurements carried out using an aberration-corrected TEM instrument (see Figure S14, Supporting Information File 1, for the RTA-annealed wafer) and are reported as dashed lines in Figure 8.

Conventional Hall effect measurements were performed on both annealed wafers and are reported in Table 1 (a scattering factor *r*_H_ = 1 was chosen in this case [20]). The results indicate that both annealing treatments yield high dopant activation, with millisecond-DSA resulting in slightly better parameters, i.e., a higher active dopant density and an overall lower sheet resistance compared to spike-RTA. Indeed, using the analysis method described in [13] and taking into account the exact location of the SiO_2_Si interfaces (as described above), we found that about 92% of the arsenic ions retained in the top Si layer are electrically active in the DSA-annealed wafer, with a maximum active concentration of ca. 1.4 × 10^20^ cm^−3^, compared to about 75% and ca. 6 × 10^19^ cm^−3^, respectively, in the RTA-annealed wafer. However, these average values do not give access to the actual dopant concentration levels in the surface region. DHE profiling was therefore used to scan the surface doping concentration in both investigated wafers.

**Table 1:** Hall effect data measured on 11 nm thick SOI wafers implanted with As^+^ (3 keV, 1 × 10^14^ cm^−3^) and annealed with spike-RTA or millisecond-laser-DSA.

annealing treatment	sheet resistance, *R*_S_ (Ω·sq^−1^)	active Hall dose, *N*_H_ (cm^−2^)	Hall mobility, µ_H_ (cm^2^·V^−1^·s^−1^)

RTA	2157	4.5 × 10^13^	65
DSA	1643	7.8 × 10^13^	49

#### Differential Hall effect

For these measurements, after each removal step (based on HF/ethanol cycle) the Van der Pauw test structures were left in a clean-room environment from one to three days, so to provide reproducible native oxide regrowth. In order to collect a maximum number of data, we performed thickness and Hall effect measurements before and after oxide stripping. However, considering the possible difference in the electrostatic configuration of the surface, i.e., the number of interface states, between samples with a stable grown oxide and samples measured just after stripping of the native oxide, and the impact of the electrostatics on the reproducibility of the Hall effect measurements (cf. Figure 4a and the related discussion), the DHE data treatment was applied separately to the two sample groups: those measured just after oxide stripping and those measured in the presence of a stable native oxide.

**Figure 9:**
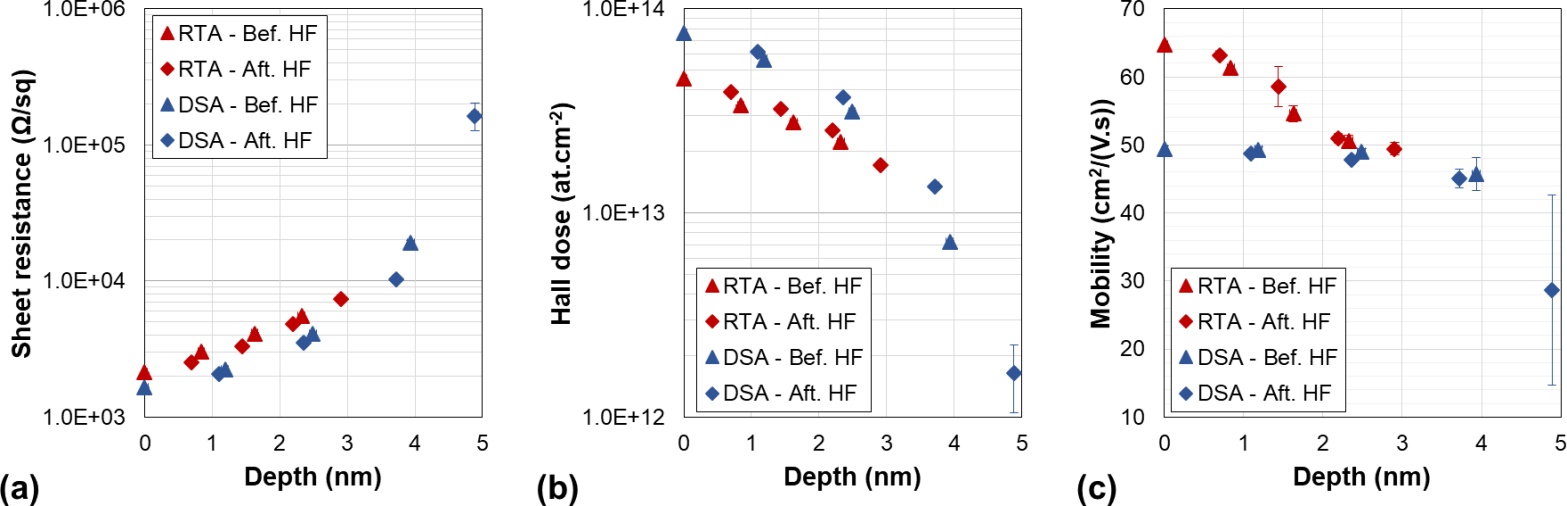
Hall effect measurements (raw data: (a) *R*_S_, (b) *N*_H_ and (c) µ_H_) of the SOI samples implanted with arsenic and annealed with DSA (red symbols) and RTA at 1050 °C (blue symbols), as a function of the etched thickness (as measured by ellipsometry).

Four successive etching processes were realised for each sample resulting in eight experimental points. All the raw Hall data from both investigated samples are reported in Figure 9. When the layer thickness decreases, the electrical parameters evolve following the expected behaviour, with the sheet resistance increasing and the active Hall dose decreasing as a function of the removed thickness. However, due to non-uniformity of the dopant distribution in depth, the observed variations are not linear. As for the carrier mobility, the RTA-annealed samples exhibits higher values than the DSA-annealed samples, in agreement with the lower active dopant concentration already inferred from conventional Hall measurements (cf. Table 1).

In addition, it has to be noted that the samples could not be profiled over the entire thickness of the active layer, as indicated by the unexpectedly high resistance value of the DSA annealed sample after 5 nm of etching (Figure 9a), and in apparent contrast with the high active fraction (>90%) of this sample (Table 1). This is attributed to the presence of the backside depletion region located at the Si/BOX interface, the impact of which on the measured values increases with the increase of the removed thickness. As reported in previous studies in similar SOI structures [18], this effect does not modify the reliability of the dopant concentration extracted at the surface, where it exhibits its maximum value. A possible solution to overcome this problem could consist in the local modification of the dopant concentration at the Si/BOX interface (for instance by a dedicated low-dose implant) so to strongly reduce the extent of the backside depletion region. However, such additional step was not considered in this work, the main focus of which is on the dopant activation at the semiconductor surface.

**Figure 10:**
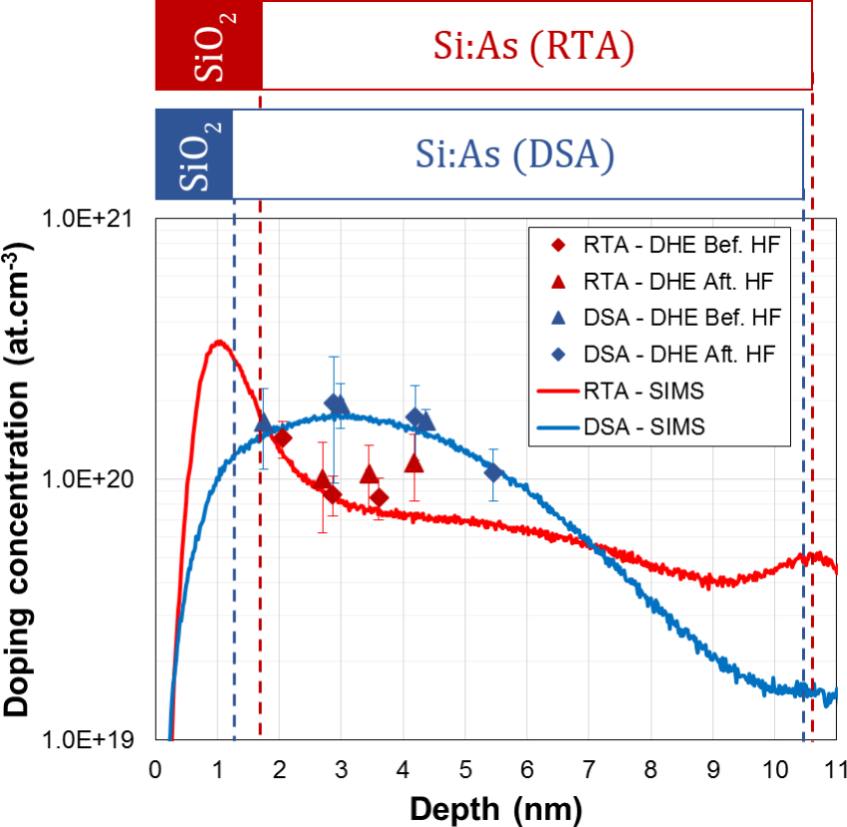
Active dopant concentration depth profiles as extracted by DHE measurements from 11 nm thick SOI wafers implanted with As^+^ (3 keV, 1 × 10^14^ cm^−3^) and annealed with spike-RTA (red symbols) or millisecond-laser-DSA (blue symbols). DHE values are compared with the corresponding arsenic chemical concentration profiles as measured by SIMS.

The calculated differential Hall values are finally presented in Figure 10. The obtained values are plotted together with arsenic concentration profiles measured by SIMS, by taking into account the actual position of the SiO_2_/top Si interface (cf. Figure 8), while corrections of the depth scale related due to the surface depletion effect have been neglected due to high doping levels measured in these samples (cf. previous section and Figure S7, Supporting Information File 1). The DHE carrier concentration profiles perfectly follow the chemical profiles measured by SIMS, confirming that both annealing methods provide a high dopant activation efficiency. More importantly, DHE measurements unambiguously show that, within the first two nanometres below the surface, millisecond annealing results in a higher active dopant concentration compared to RTA, making DSA a better candidate than RTA for contact resistance reduction in future FDSOI technologies.

## Conclusion

In this paper, we presented an enhanced differential Hall effect measurement method that allows to determine, with sub-nanometre resolution, the level of dopant activation close to the surface for Si and SiGe. In the case of SiGe, which constitutes the most challenging process, we showed the reliability of the SC1 chemical solution thanks to its slow etch rate, stoichiometry conservation and low roughness generation. For both materials, our method include a direct measurement of the removed thickness after each removal step, so to avoid averaging the etch rate and improve the accuracy of calculated values. Then, we demonstrated the reliability of a complete DHE procedure, with an etching step as small as 0.5 nm, on a dedicated 20 nm thick SiGe test structure fabricated by CVD and uniformly doped in situ with boron during growth.

The developed method was finally applied to the investigation of dopant activation achieved by advanced annealing methods in two material systems: 6 nm thick SiGeOI and 11 nm thick SOI. In the first case, we showed that a doping process based on nanosecond-laser annealing can be successfully applied to ultrathin SiGeOI layers, with achieved active dopant concentrations at the surface well above 1 × 10^20^ cm^−3^, which is a promising result in view of improving contact resistivity in SiGe source/drain regions of advanced devices. In the second case, DHE measurements unambiguously show that, within the first few nanometres below the surface, millisecond-laser-DSA can result in a higher active dopant concentration compared to RTA, making DSA a better candidate than RTA for contact resistance reduction in future FDSOI technologies. In summary, thanks to the improvements implemented in this work, DHE is shown to be a unique sensitive characterisation technique for a detailed investigation of dopant activation in ultrashallow layers, providing sub-nanometre resolution for depth profiles of both dopant concentration and carrier mobility.

## Supporting Information

File 1 Additional experimental data.
