# Supplementary material for: A differential Hall effect measurement method with sub-nanometre resolution for active dopant concentration profiling in ultrathin doped Si1−xGex and Si layers
Source: Beilstein J Nanotechnol. 2018 Jul 5;9:1926–39. doi: 10.3762/bjnano.9.184 (PMC6036972; doi:10.3762/bjnano.9.184)
Supplement: File 1 — Additional experimental data. [file Beilstein_J_Nanotechnol-09-1926-s001.pdf]

Supporting Information

for

A differential Hall effect measurement method with sub-nanometre resolution for active dopant concentration profiling in ultrathin doped $Si_{1-x}Ge_x$ and Si layers

Richard Daubriac*[1], Emmanuel Scheid[1], Hiba Rizk[1], Richard Monflier[1], Sylvain Joblot[2], Rémi Beneyton[2], Pablo Acosta Alba[3], Sébastien Kerdilès[3] and Filadelfo Cristiano[1]

Address: [1]LAAS-CNRS and Univ. of Toulouse, 7 avenue du Colonel Roche, 31400 Toulouse, France; [2]STMicroelectronics, 850 rue Jean Monnet, 38926 Crolles, France and [3]CEA-LETI and Univ. of Grenoble, 17 rue des Martyrs, 38054 Grenoble, France

Email: Richard Daubriac* - richard.daubriac@laas.fr

* Corresponding author

Additional experimental data

X-ray diffractometry measurements

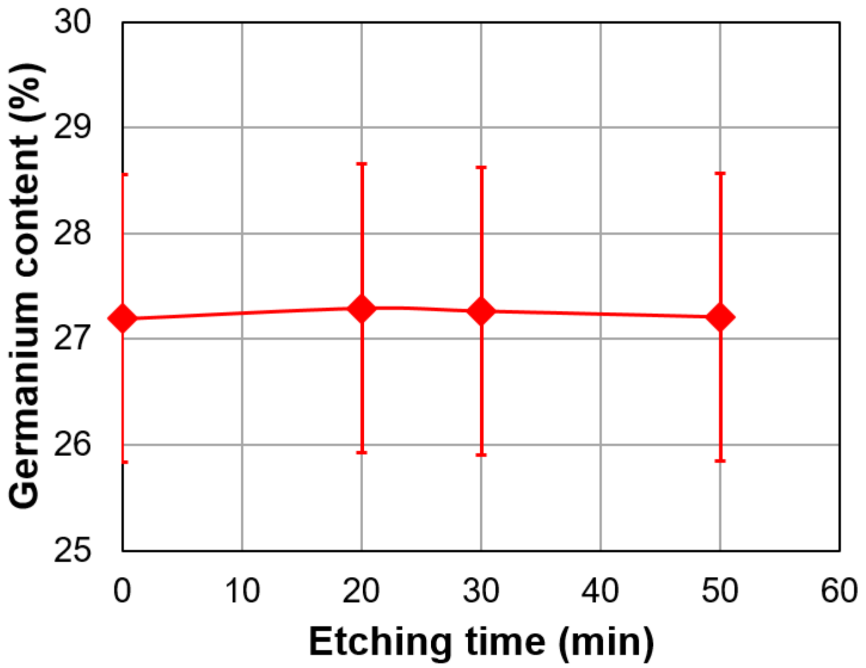

Figure S1: Germanium content as function of etching time for a $Si_{0.7}Ge_{0.3}$:B/Si system etched with SC1. After 50 min of etching time with SC1 solution, the original germanium content remains constant (x_{Ge} = 0.27).

Tapping mode atomic force microscopy characterisations

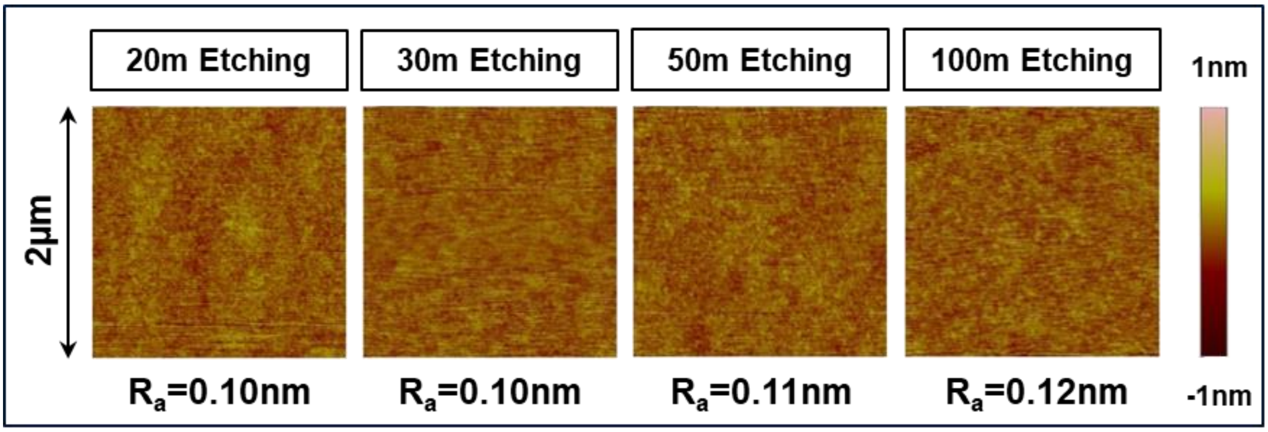

Figure S2: Roughness measured by AFM for different etching times with SC1 for a $Si_{0.7}Ge_{0.3}$:B/Si system. For etching times between 20 and 100 min, the arithmetic average R_a varies from 0.10 to 0.12 nm. These values show that SC1 solution induces low roughness.

Hall encapsulation cell images

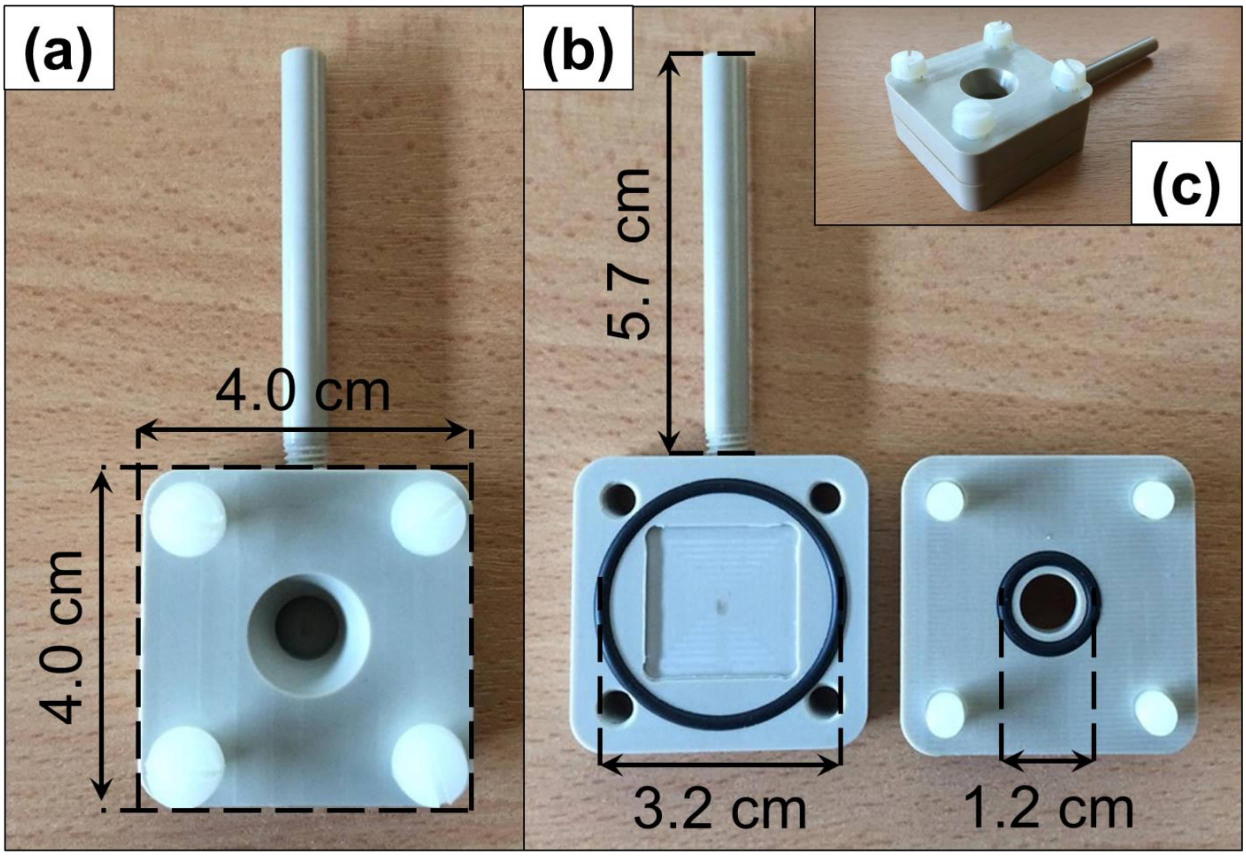

Figure S3: Hall encapsulation cell designed to protect metallic contacts during etching of SiGe and Si. (a) Top view, (b) inside view and (c) global view. All materials for the designed Hall cell have been chosen to resist HF + ethanol and SC1 chemistry: Teflon for screws, Viton for the O-rings and PEEK for cell body.

Van der Pauw structures mask design

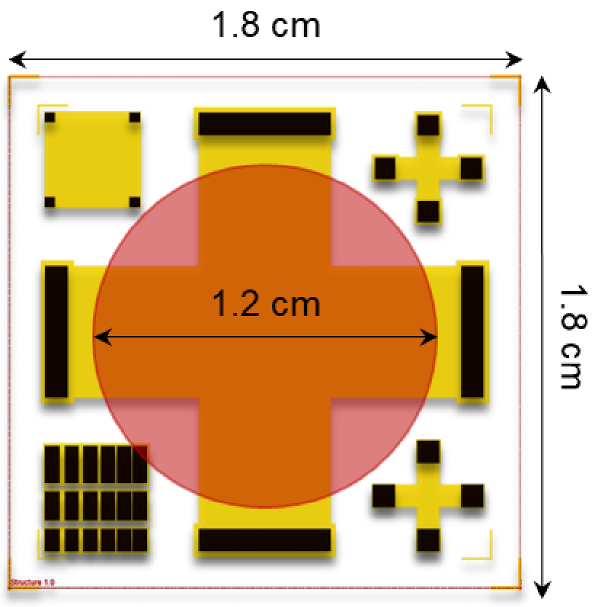

Figure S4: Mask used for fabrication of Van der Pauw test structures. The central structure is used for both conventional and differential Hall effect measurements (red circle defines the region where the etching process occurs in the Hall encapsulation cell). Peripheral structures are TLM (bottom, left corner) for contact resistance measurements and Van der Pauw structures (square- and cross-shaped structures) for conventional Hall effect measurements only.

Sheet resistance and Hall coefficient measurements

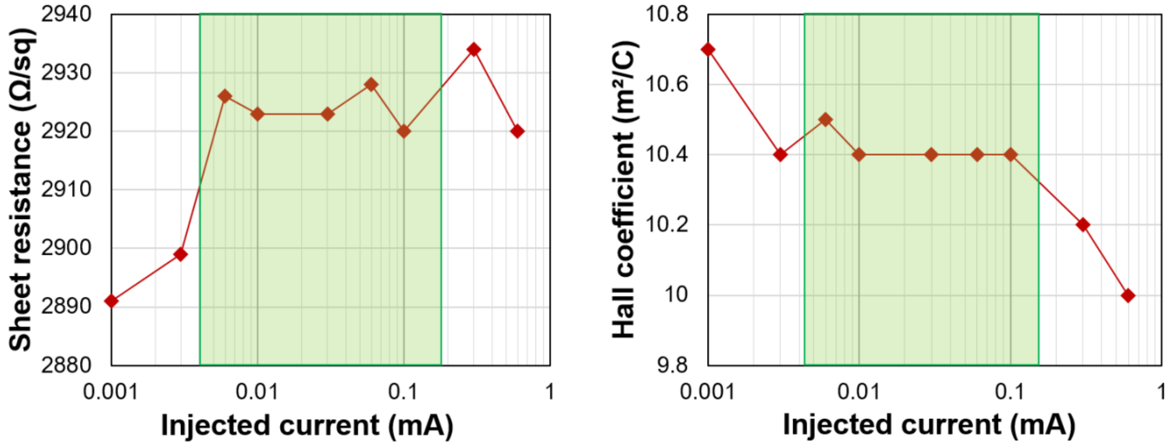

Figure S5: Sheet resistance and Hall coefficient as functions of the injected current for a 20nm thick $Si_{0.77}Ge_{0.23}$:B layer. These graphs illustrate a region of high stability of sheet resistance R_S and Hall coefficient R_H for injected currents between 0.006 mA and 0.1 mA. Thanks to this region of high reproducibility, errors on differential values are minimized.

Hall scattering factor values

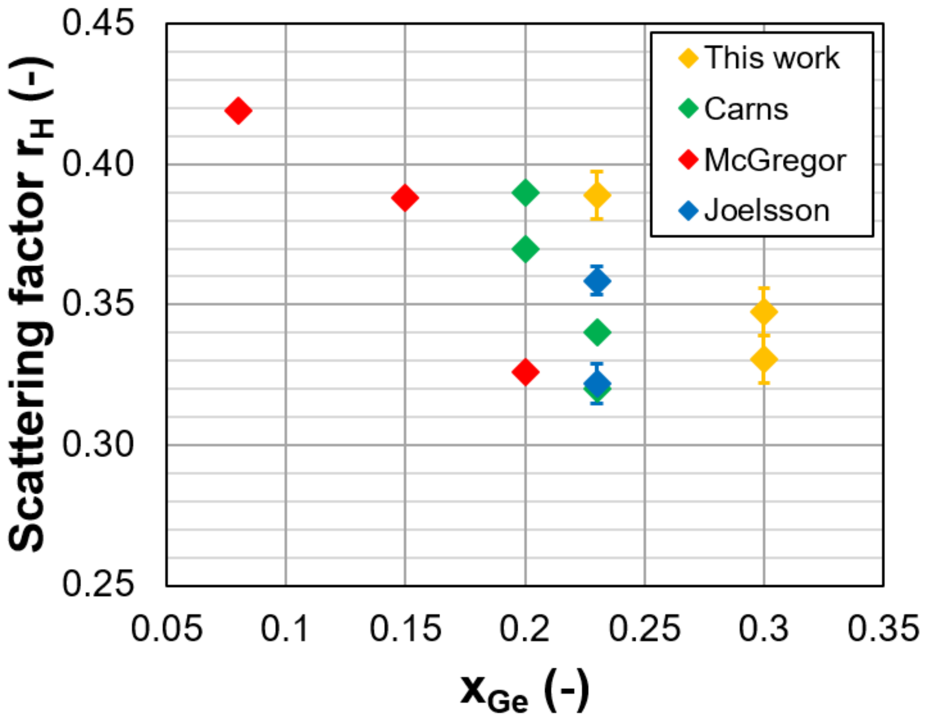

Figure S6: Scattering factor of boron-doped SiGe as a function of the germanium content. Scattering factors found through conventional Hall effect measurements range from 0.35 to 0.4 for, respectively, germanium contents of 30 atom % and 22 atom % [1-3].

Depletion-width calculations

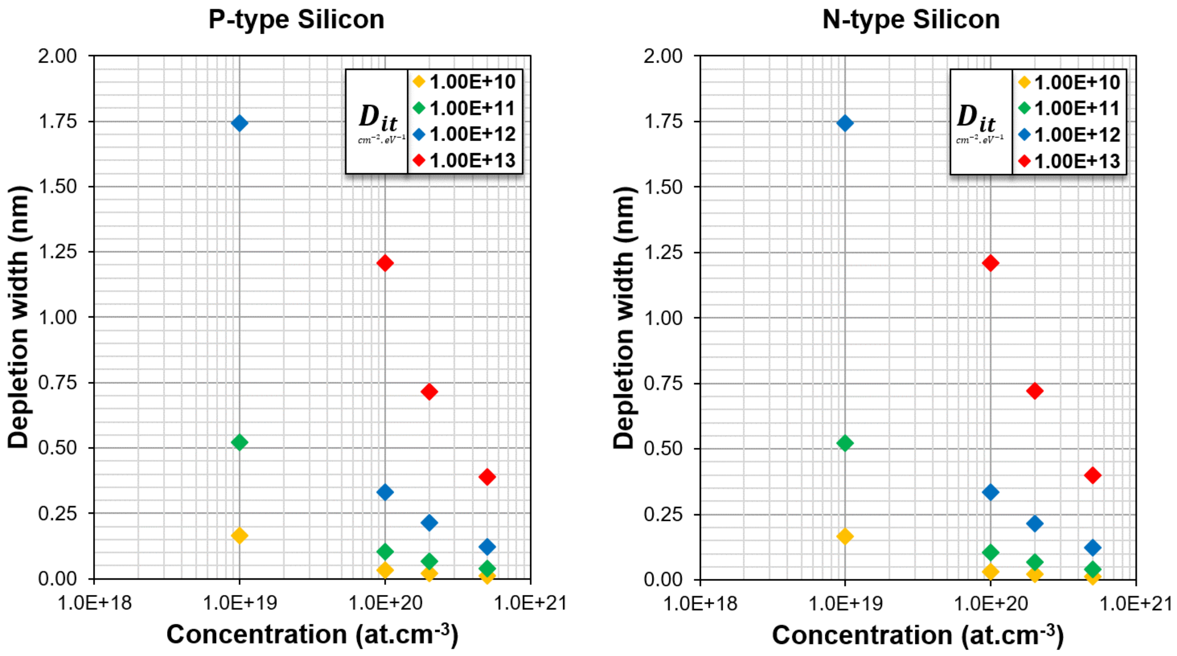

Figure S7: Depletion width as a function of the concentration for p-type and n-type silicon. These values have been obtained using TCAD Sentaurus simulations (solving the Poisson equation at the interface of an oxide of 2 nm, with a trap charge density D_{it}, and a uniformly doped silicon bulk) [4,5].

Secondary ion mass spectrometry profiling

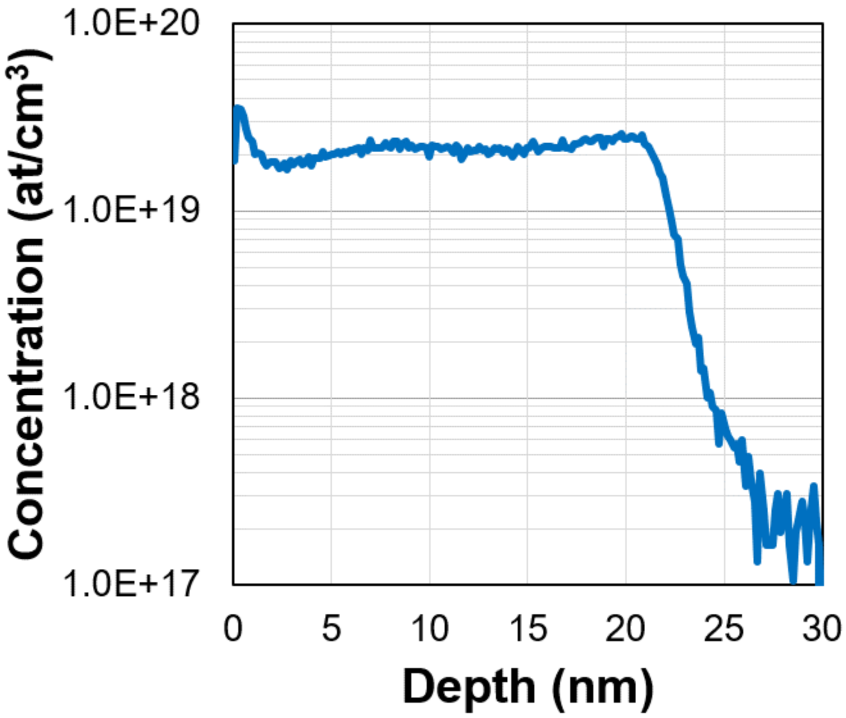

Figure S8: SIMS profile of boron in the investigated 20 nm $Si_{0.7}Ge_{0.3}$:B test structure. SIMS shows a uniform boron profile of around 10^{19} cm^{-3}. This fully electrically active test structure has been used to validate the differential Hall effect measurement method developed in this work.

Laser thermal annealing process for Coolcube application

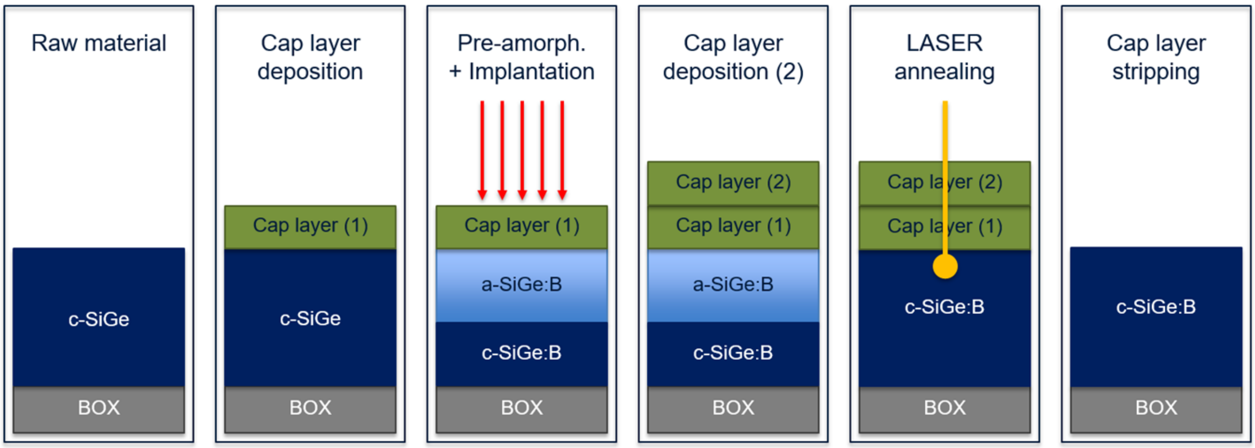

Figure S9: Process steps used to fabricate the investigated 6 nm $Si_{0.75}Ge_{0.25}OI$:B layers. Two steps are necessary to obtain a 6 nm boron-doped $Si_{0.75}Ge_{0.25}OI$ layer. First, a 3 nm nitride deposition followed by Ge and B implantation (germanium for amorphisation and boron for doping). Then, a second 3 nm nitride layer is deposited before laser annealing at different energy densities. Finally, nitride capping layers are stripped.

Transmission electron microscopy images

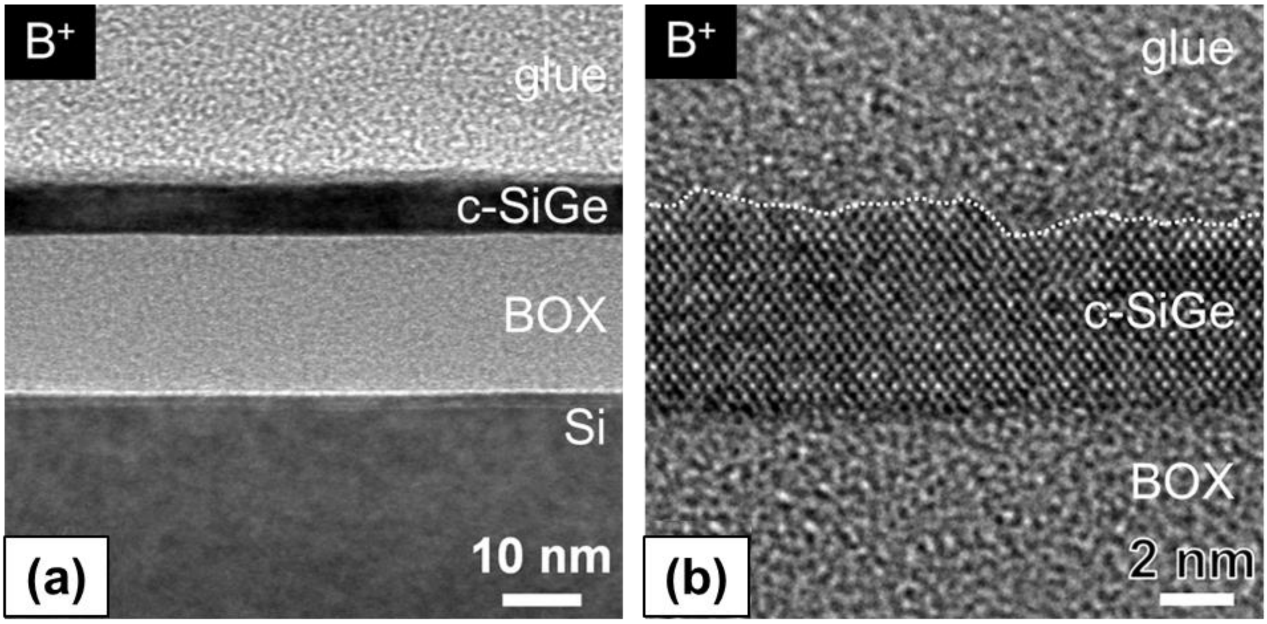

Figure S10: (a) TEM and (b) HRTEM micrographs of the as-implanted 6 nm $Si_{0.75}Ge_{0.25}$OI:B layer. (a) shows the three-layer stack: SiGe/BOX/Si. (b) indicates irregularities at the SiGe surface suggesting that Ge implantation parameters did not yield a continuous amorphous surface layer.

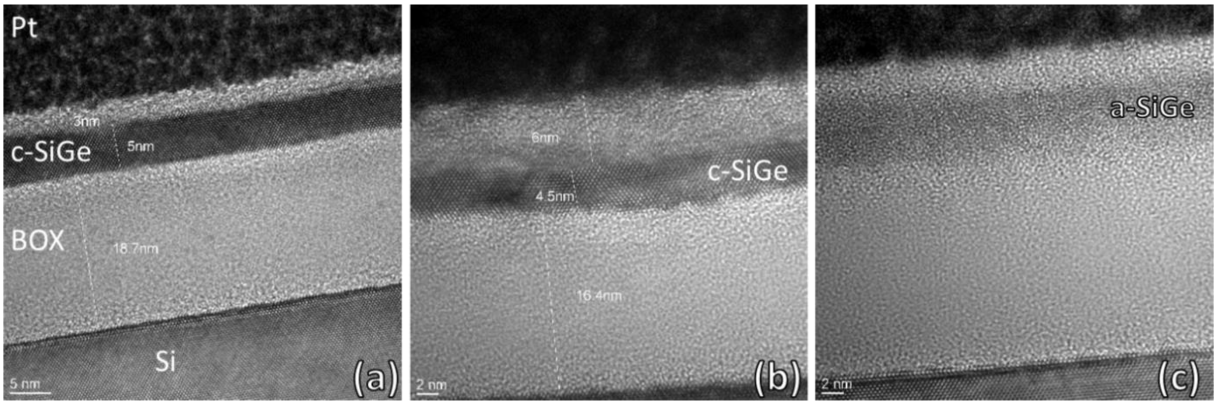

Figure S11: TEM images of $Si_{0.75}Ge_{0.25}$OI:B layers annealed at different LTA energy densities: (a) 0.74 J·cm^{-2}, (b) 0.76 J·cm^{-2} and (c) 0.79 J·cm^{-2}. The presented images demonstrate two different states of annealing: (a) and (b) "no melt" regime; panel (c) "full melt" regime. For (a) and (b), the crystal quality is preserved although some interface roughness appears in (b); for (c), "full melt" is obtained and the layer becomes amorphous.

Spreading resistance profiling

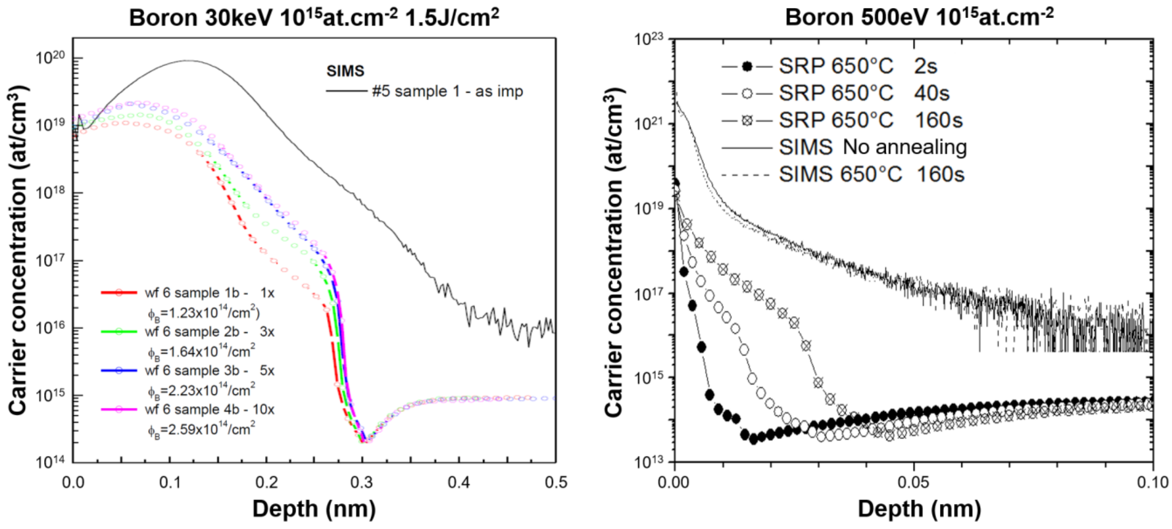

Figure S12: SRP profiles indicating dopant activation close to the surface for low thermal budget anneals. (left) Experimental results from SRP (empty circles) measurements for Si samples implanted with boron ions (non-amorphising conditions) and laser annealed with a “non-melt” energy density of 1.5 J·cm^{-2} for different number of pulses (1, 3, 5 and 10). One of the SIMS profiles measured after LTA (dark line) is also shown. No diffusion occurs during anneal. (right) SRP (symbols) and SIMS (lines) profiles of samples implanted with boron at 0.5 keV and annealed at 650 °C for different times (2, 40 and 160 s).

Tapping mode atomic force microscopy characterisations

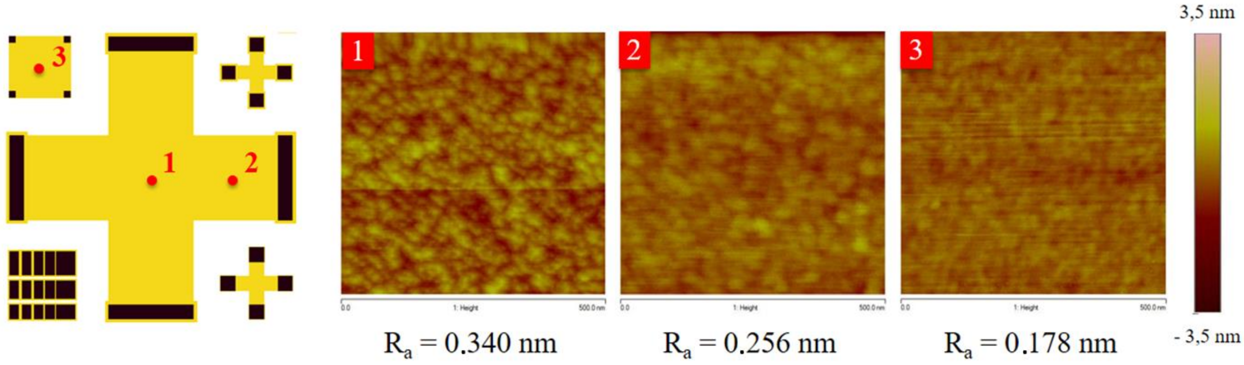

Figure S13: AFM images of the $Si_{0.75}Ge_{0.25}$OI:B layer etched for a time of 90 min and annealed at 0.68 J·cm^{-2}. Three different regions are scanned by AFM: (1) etched zone (centre of the Van der Pauw structure); (2) periphery of the etched zone (close to the protecting O-ring); (3) non-etched zone (protected during the etch process). The R_a value in the centre of the Van der Pauw structure is negligible compared to the whole etched thickness after 90 min of SC1 chemistry (1.3 nm).

STEM-EDX characterisations

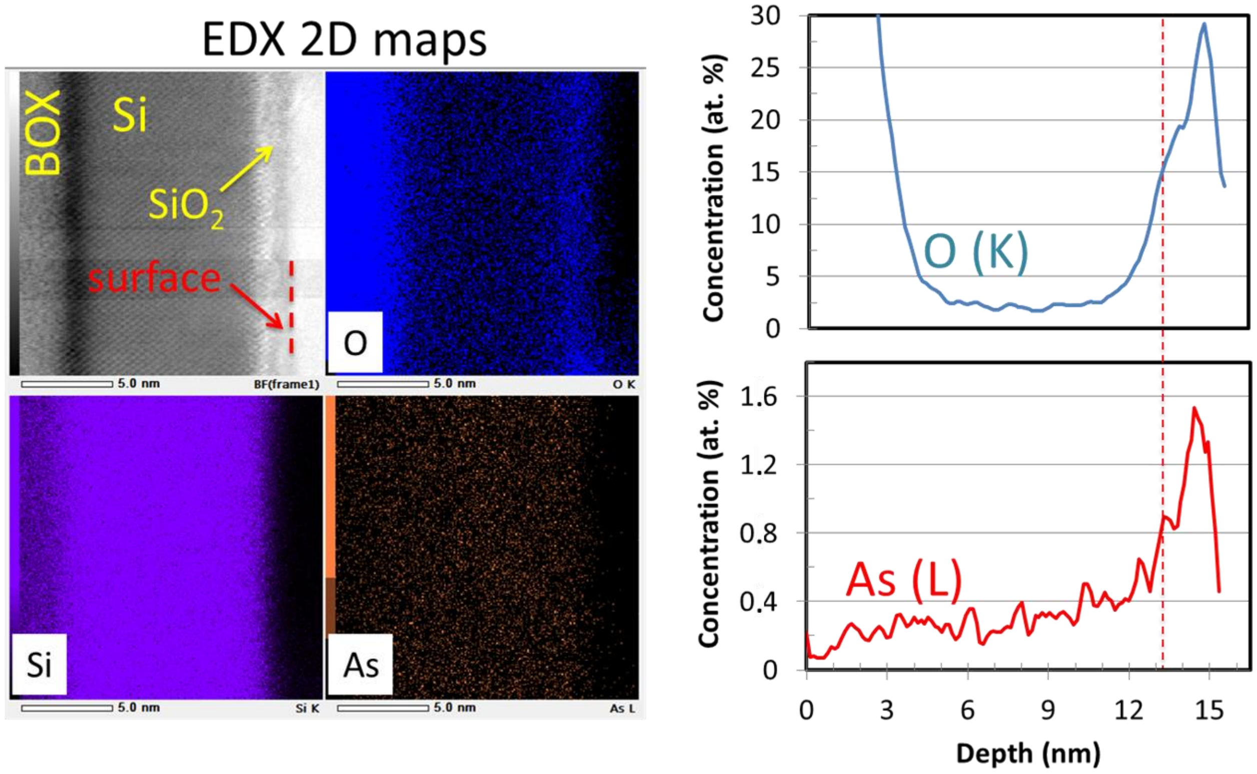

Figure S14: (left) STEM-EDX image and O, Si and As maps of the 11 nm SOI:As layer annealed by RTA. (right) Quantified concentration profiles of arsenic and oxygen. The obtained profiles are in agreement with the respective SIMS profiles and confirm the position of SiO_2/Si interfaces.

References

1. Carns, T. K.; Tanner, M. O.; Wang, K. L. *J. Electrochem. Soc.* **1995,** *142,* 1260–1266. doi:10.1149/1.2044161
2. McGregor, J. M.; Manku, T.; Noël, J.-P.; Roulston, D. J.; Nathan, A.; Houghton, D. C. *J. Electron. Mater.* **1993,** *22,* 319–321. doi:10.1007/BF02661384
3. Joelsson, K. B.; Fu, Y.; Ni, W.-X.; Hansson, G. V. *J. Appl. Phys.* **1997,** *81,* 1264–1269. doi:10.1063/1.363906
4. Yeo, Y. K.; Hengehold, R. L. *J. Appl. Phys.* **1987,** *61,* 5070–5075. doi:10.1063/1.338331
5. TCAD Sentaurus Process User Guide, I-2013.12 version, Synopsis, Inc.: Mountain View, California, USA, 2013.
